# Choose Ye Which

**Published:** 1877-07

**Authors:** Ben. H. Pratt


### THE BISTOURY.

Vol. XIII. ELMIRA, N. Y., JULY, 1877. No. 2.

### Contributions.

### CHOOSE YE WHICH.

BEN. H. PRATT.

Real modesty is the crowning glory of a woman, the guinea stamp of a man's nobility, and that which in childhood is more beautiful than beauty. It is so precious a thing that he who has it not, would fain affect it, and she who does not possess it, must needs counterfeit it. It is one of the virtues that no one can put on and lay off at will. Its roots are in the conscience, and its tendrils interosculate throughout every sentient atom of the individuality. It is not the boon of innocence, nor does ignorance possess it in a higher degree than wisdom. It is oftener the child of wise teaching and the offspring of upright example. The parent cannot too early begin to plant within the heart of childhood the seeds of modesty, and it is the mother's province, from day to day and from year to year, to so nurture and train the growth that may ensue, until it shall have acquired a strength and vigor that no winds of temptation may blast, and no drouth of adversity kill.

Parents make no greater mistake than in supposing that if they can only keep their children aloof from the knowledge of that which is bad until a period of life when a certain maturity is supposed to give moral strength—the young will have passed the most dangerous crisis of their lives, and thenceforth be comparatively safe from contamination. They guard with strictest care the ear and eye of childhood, lest a foul word or an immodest act reveal the existence of depravity in the world. They impart nothing but the purest teachings, and while they continually hold up to the view of their children the purest examples, and strive to lead them by the purest precepts through paths of virtue, they are unwittingly deceiving them into believing that there is no evil in the world. But the day upon which these innocent ones discover that there is in the midst of this beautiful paradise of parental teaching, a tree upon which hangs temptingly, beautiful but forbidden fruit, the halo that erst overspread all things mundane disappears—vanishes into thin air, and the glamour of life is dispelled. And such a time comes into the life of every child that lives long enough to discern the difference between good and evil, and when it comes, there also comes a knowledge of parental deception—a conviction that the world is not such as it had been pictured—that there is more for them to do in life than to pursue the pleasant paths of virtue, and a far more difficult task is set them in avoiding the evil with which that path is beset. The shock is all the greater in that it is so sudden and unexpected, and the glittering lure that presents itself is all the more dazzling by reason of its novelty. This is the danger point in the life of every young person, and we are compelled to say that the number of those who are so tutored in ignorance of evil and fall beneath its temptation is greater than that of those who, knowing the evil, successfully combat and overcome it.

If a joiner should essay to teach a boy his trade, we would deem him a very unwise teacher if all through his apprentceship the learner were given material to work upon which was wholly free from knots and flaws and cross-grain. Such an one, so taught, having completed his apprenticeship and set himself up as a journeyman, would find himself wholly unprepared to enter the shops with those who had been thoroughly initiated into the mysteries of overcoming cross-grains and knots and woody perversities of every description. He must either submit to the mortification of re-learning his trade or abandon all the benefits of what he had learned for an occupation with which he is wholly unfamiliar. Conceive the discouraging effects of such training, and the fatal disappointment of a discovery like this.

The true way to teach modesty in the young is to acquaint them with that which is immodest as well as with that which is modest. It is grossly wrong to conceal from the young the fact that there is evil in the world. They should know what they must meet with when they arrive at that stage in life when the temptation comes to them. They can combat it the better if its mockeries and inherent weaknesses are known and the effects of yielding thereto are previously pointed out. Parents should be familiar with their children upon all subjects, and especially upon those on which so surely depend their children's virtue. That is, therefore, a mistaken policy, and one which is pursued by so many parents, which excludes from the family circle such books as tend to instruct children in the ways of the world. Plainly written books by authors above reproach, and disseminated with a view to instruct rather than to entertain and excite—books which fearlessly call sin by its right name and hold up its hideousness to view—these are often discarded by the head of the family because they fear lest such may set the young to thinking upon impure things in an impure way. Perhaps it would be better for a boy or girl never to know to what degradation the world has come, provided it were possible to hide it from them forever. But this is impossible, and the question for every parent to consider is this: Which is better, that my son or my daughter learn these things in my own house, seated around my own hearth, surrounded by the moral influence of home proprieties, under my own eye, with entire freedom to ask explanations from me and converse with me upon the subjects treated, and where I can at once correct any tendency to pursue the theme in an impure direction; or, to forbid the presence of such books, and learn, when it is too late, that this boy or this girl, having been denied the perusal of really profitable works upon vital subjects and couched in proper language, has clandestinely sought and found and then devoured the foul contents of those obscene books with which the land is cursed—devoured them perhaps in improper places, among improper people, and with no check upon the fancy whatever? It is the alternative that the parent must consider, for in nine cases out of ten, if one is denied the other is attained to by the thoughtlessness of youth and the depravity which is innate in every juvenile heart.

Another great mistake made by parents is in ruling out of the family the newspaper, because it must, in the ordinary routine of news giving, contain much that is falsely deemed improper for young people to read. Why, improper? To this question the writer once received this reply: "Because, my children read these items of news, and not thoroughly understanding their meaning, ask me questions which I cannot answer without going into explanations upon subjects which I decidedly object to discuss in my family before my childen." Here is the trouble. He, like many other well meaning but dangerously injudicious parents, proposed to keep his children in utter ignorance of the existence of wickedness in the world until they found it out for themselves. What then? They will either despise the home teaching that kept them in such ignorance, or seek elsewhere and in improper places for that which would have had no special fascination but for the fact that it was a novelty, and had become doubly attractive because forbidden at home. And as there is no medium of information that will compare with the newspaper, the too prudish parent will sacrifice his children's intelligence to his mistaken notions of the true method of morally rearing his family. Just as men and women must think much which it would not be proper to converse upon, so the newspaper must necessarily contain much which all should know, but which need not be read aloud or discussed. There is a wide distinction between the journal that gives all the news, both the proper and the improper sort, and that class of periodical literature which aims, by dwelling upon the vulgarly sensational, to pervert the public taste and create a demand for that obscene stuff which is the stock in trade of a certain class of publications. That newspaper is the safest in the family which details, in proper language, the events of the time; and those prudish milk-and-water editors who affect a modesty they have not, by either omitting everything that departs from the conventional, or refusing thereto in language more vulgar in its attempts to be nice than if it were outspokenly and honestly plain, deserve especial approbrium.

In fine, children should be taught what is evil, that they may avoid it, as well as that which is good in order that they may seek after the good. Parents, early begin to cultivate the confidence of your children. Let them know that in you they have a thorough sympthizer as well as a safe adviser. Teach them to withhold from you no knowledge of wrong, however wrong, they may possess, and thus you will be enabled many times to say that word which is oftentimes the savior of their virtue. Do not make children think that they must not tell you anything because it is too bad to tell. There is nothing too bad for a child to go to a parent with, provided the parental heart be pure, and maintains a sincere desire to "consider all things and hold fast to that which is good."

